# Comparison of TiF_4_, CPP-ACP, and NaF in preventing demineralization in irradiated bovine enamel and dentin *in vitro*

**DOI:** 10.1590/1678-7757-2024-0524

**Published:** 2025-05-30

**Authors:** YAO Lin, LI Yanyao, FU Di, JI Mengzhen, ZOU Ling, Li JIANG

**Affiliations:** 1 Sichuan University West China Hospital of Stomatology State Key Laboratory of Oral Diseases Chengdu China Sichuan University, West China Hospital of Stomatology, State Key Laboratory of Oral Diseases & National Center for Stomatology & National Clinical Research Center for Oral Diseases & Department of Conservative Dentistry and Endodontics, Chengdu, China.; 2 Sichuan University West China Hospital of Stomatology Department of General Dentistry Chengdu China Sichuan University, West China Hospital of Stomatology, Department of General Dentistry, State Key Laboratory of Oral Diseases, National Clinical Research Center for Oral Diseases, Chengdu, China.

**Keywords:** Radiation-related caries, Demineralization, Titanium tetrafluoride, Sodium fluoride, Casein phosphopeptide-amorphous calcium phosphate

## Abstract

**Objectives:**

To investigate the effects of sodium fluoride (NaF), casein phosphopeptide-amorphous calcium phosphate (CPP-ACP), and titanium tetrafluoride (TiF_4_) on the prevention of demineralization in irradiated bovine enamel and dentin *in vitro* .

**Methodology:**

The enamel and dentin sample blocks were subjected to 50 Gy of radiation and divided into five groups (n=13): the deionized distilled water group, the NaF group, the CPP-ACP group, the NaF + CPP-ACP group, and the TiF_4_ group. After being treated with various materials for 30 minutes, the samples were remineralized for 12 hours and demineralized for 48 hours. The samples were then evaluated by scanning electron microscope (SEM), atomic force microscope (AFM), energy dispersive spectrometer (EDS), and transverse microradiography (TMR). Data were analyzed by ANOVA and the Kruskal-Wallis H test (α=0.05).

**Results:**

SEM and TMR indicated that the TiF_4_ group promoted more mineral deposits on the enamel and dentin samples, showing the least mineral loss and the lowest lesion depth. AFM results showed that the NaF + CPP-ACP group had the lowest enamel roughness (p<0.05), whereas the TiF_4_ group showed the lowest roughness in dentin samples (p<0.05). EDS showed that titanium (Ti) was deposited on the surface of the TiF_4_ group samples, whereas the NaF + CPP-ACP group more greatly aggregated fluorine.

**Conclusion:**

TiF_4_ significantly impacted the prevention of demineralization in irradiated dental hard tissues. Combining NaF and CPP-ACP more effectively prevented demineralization than either agent used alone.

## Graphical abstract

**Figure:**
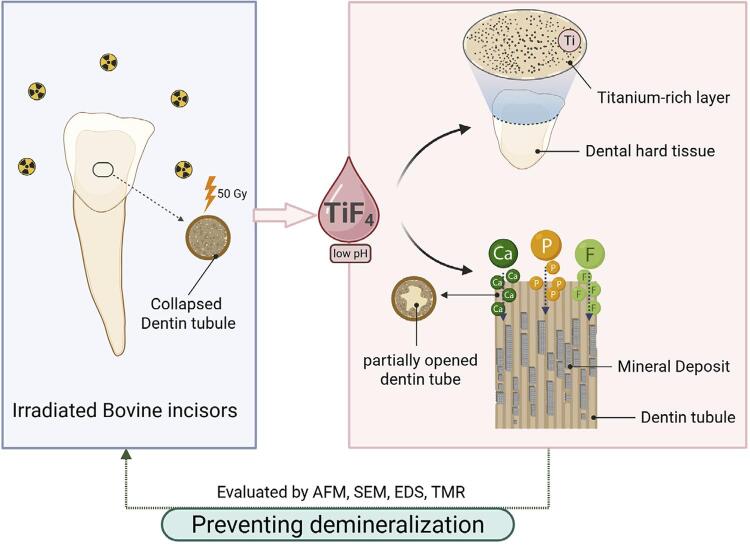

## Introduction

Head and neck cancer (HNC) configures a prevalent malignancy associated with significant morbidity and mortality, representing a considerable global public health challenge.^1 - 3^ Although surgery has consistently played a primary role in treating HNC,^4^ radiation therapy often serves as either a primary or adjuvant treatment for HNC due to the complex anatomy of the head and neck.^5^ However, radiotherapy for HNC can lead to complications and adverse reactions in oral tissues,^6^ such as infection, xerostomia, radiation-related caries (RRC), and osteonecrosis.^7^

RRC constitutes an aggressive disease that can develop after radiotherapy for HNC, with a prevalence of approximately 30% among patients undergoing this treatment.^8 , 9^ It frequently affects the smooth surfaces of the teeth (which are typically less susceptible to cariso) and the mandibular incisors.^10^ Previous studies have shown that radiotherapy can directly damage dental hard tissues.^11 - 13^ For example, radiation could induce chemical reactions that might weaken the inter-crystalline bonds in the enamel structure. Likewise, radiation-induced degradation of the organic matrix and changes in mineral composition may reduce dentin hardness and toughness. Besides, radiotherapy causes dysfunction of the salivary glands, causing insufficient saliva and significantly increasing the risk of RRC.^14^ As a result, RRC progresses rapidly and affects multiple teeth, complicating restoration efforts and resulting in a high rate of restorative failure, which leads to significant treatment costs.^15^ Furthermore, extracting residual tooth roots may increase the risk of osteoradionecrosis.^16^ Therefore, prioritizing the prevention of RRC is crucial for enhancing the quality of life for patients undergoing radiotherapy for HNC.^17 , 18^

A variety of chemical and biological agents have been investigated as minimally invasive strategies to prevent and control dental caries. Topical applications of fluoride are most commonly used to prevent RRC, with sodium fluoride (NaF) being one of its most prominent forms.^7 , 19^ The annual application of sodium fluoride varnish has been reported to reduce the incidence of RRC by 64%.^20^ Recently, it has been discovered that titanium tetrafluoride (TiF_4_) could form a durable titanium-rich layer on the surface of teeth, serving as an acid-resistant barrier against enamel erosion.^21^ NaF and TiF_4_ have shown significant efficacy in reducing mineral loss associated with radiation-induced dentin caries *in vitro* .^22^ Comar, et al.^23^ (2017) found that 4% TiF_4_ varnish showed a superior remineralization effect on enamel caries than NaF varnish. Therefore, TiF_4_ may more effectively prevent RRC than NaF.

Casein phosphopeptide-amorphous calcium phosphate (CPP-ACP) configures a biological anti-caries agent derived from milk casein that has shown a long-term remineralization effect on early caries lesions.^24^ Besides, CPP-ACP can interact with fluoride ions to improve the prevention of demineralization and promote remineralization.^25^ The combination of NaF and CPP-ACP has been shown to inhibit the progression of root caries, restoring the hardness of affected areas and reducing the size of the lesions.^26^ However, no studies have explored whether CPP-ACP interferes with the demineralization of enamel and dentin following radiotherapy. Thus, the use of NaF and CPP-ACP in preventing and treating RRC warrants further investigation as a potential new option.

This study aimed to investigate the effects of NaF, CPP-ACP, NaF with CPP-ACP, and TiF_4_ on the prevention of cariogenic demineralization in irradiated enamel and dentin. Scanning electron microscope (SEM), atomic force microscope (AFM), energy dispersive spectrometer (EDS), and transverse microradiography (TMR) assessed the surface micromorphology, surface roughness, elemental content and distribution, and lesion depth of dental hard tissues, respectively.

This study hypothesized that TiF_4_ and combining NaF and CPP-ACP could effectively prevent the demineralization of enamel and dentin after radiotherapy.

## Methodology

**Figure 1:**
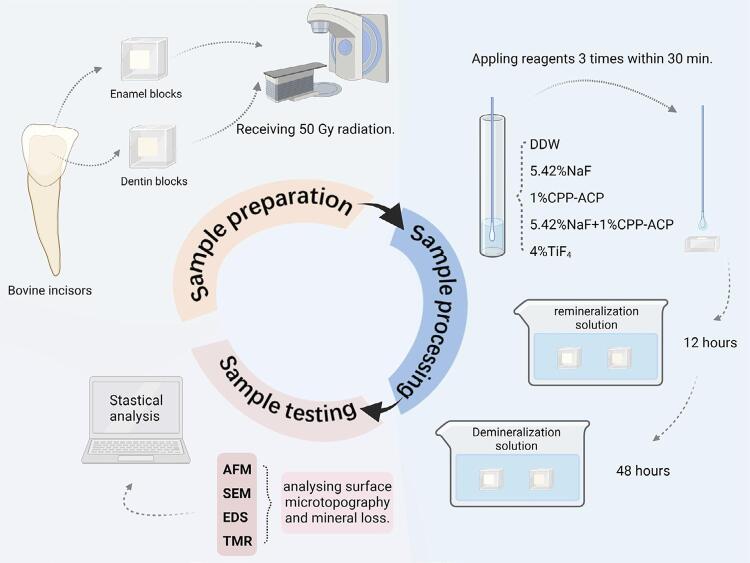
The flowchart of the experimental procedure (created with BioRender.com).

The experimental method flow is shown in Figure 1 .

### Sample preparation

The required sample size was estimated a priori on G*Power 3.1 based on one-way ANOVA parameters. According to Parisay, et al.^27^(2024), the input parameters included effect size (f=0.5), power (β=0.8), and alpha error (p=0.05). The calculation indicated a minimum of 11 sample units per group. To account for potential laboratory errors, 13 samples were prepared in each group, resulting in a total of 65 samples.

Incisors from five-year-old cows — purchased from a local slaughterhouse — were used in this study. Bovine incisors with uniform crown sizes free from cracks or white spots were selected for the experiments. The permanent bovine incisors were immersed in a 0.1% thymol solution (Macklin, Shanghai, China).^21^ Subsequently, the soft tissues and debris on the surface were removed using surgical instruments and an ultrasonic cleaner (FS20; Fisher Scientific Co., Pittsburgh, USA). Intact bovine teeth were sectioned into uniform enamel and dentin blocks using a slow precision cutting machine (Minitom; Struers, Copenhagen, Denmark) and an emery cutting saw blade (EXAKT300; EXAKT, Norderstedt, Germany) under a continuous flow of deionized water. The prepared enamel and dentin blocks were embedded in polymethyl methacrylate (Macklin, Shanghai, China) within a square mold with dimensions of 1 cm × 1 cm. The sample blocks were then ground and polished with 300-, 800-, 1200-, and 1500-grit^28^ waterproof silicon carbide paper (Yu Ying, Foshan, China) using water-cooled carborundum discs (Struers Minitom; Struers, Copenhagen, Denmark), ensuring that the surfaces of the enamel and dentin were exposed to at least 4×4 mm^2^. Finally, 65 enamel samples and 65 dentin samples were obtained, soaked in a PBS solution, and stored in a refrigerator at 4 °C.

### Sample irradiation

The medical linear accelerator (Synergy; Elekta, Stockholm, Sweden) was used to irradiate the samples. The irradiation distance was set at 100 cm from the window surface, with an irradiation field measuring 20×20 cm^2^ and a dose rate of 2.5 Gy/min. Fractionated radiation was employed and all samples remained within the pre-established radiation field range. To prevent machine overload, each sample was irradiated for two minutes, followed by a cooling period of 10 minutes before the next round of irradiation. The enamel and dentin samples received a total dose of 50 Gy by carefully controlling the irradiation time.^29 , 30^

### Materials application

Enamel and dentin samples were randomly divided into five groups (n=13) and treated as in Table 1 .

**Table 1:** Group of experiments

Groups	Reagent solutions	Application time
DDW group	DDW	30 minutes
NaF group	5.42% NaF (Macklin, Shanghai, China)	
CPP-ACP group	1% CPP-ACP (Macklin, Shanghai, China)	
NaF + CPP-ACP group	5.42% NaF and 1% CPP-ACP	
TiF_4_ group	4% TiF_4_ (Macklin, Shanghai, China)	

NaF, CPP, ACP, and TiF_4_ were provided by Macklin (Shanghai, China) as powdered chemicals. In the experiment, these substances were dissolved in deionized distilled water (DDW) to prepare reagent solutions of 5.42% NaF (pH=7.82), 1% CPP-ACP (pH=6.93), and 4% TiF_4_ (pH=1.74).^31 , 32^ Specifically, the CPP-ACP solution was prepared by mixing equal volumes of the 1% CPP and 1% ACP solutions. These materials were applied to the sample surface three times using micro brushes (Jaan, Guangzhou, China), and each application was repeated after a 10-minute interval. This procedure ensured that the sample surface remained moist for a total of 30 minutes. Subsequently, the samples were rinsed with DDW for 2 minutes and then placed in a remineralization solution (20 mM HEPES, 0.9 mM KH_2_PO_4_, 1.5 mM CaCl_2_, 130 mM KCl, pH 7.0) at 37 °C for 12 hours.^33^ After this period, the surface was washed with DDW for an additional 2 minutes and placed in a demineralization solution (2.2 mM KH_2_PO_4_, 2.2 mM Ca(NO_3_)_2_, 5.0 mM NaN_3_, 0.5 ppm NaF, 50 mM acetic acid, pH 4.5) at 37 °C for 48 hours.^34 , 35^ Finally, all demineralized samples were rinsed with DDW for 2 minutes and dried in preparation for subsequent experiments.

#### AFM observation

Following the aforementioned treatment, enamel and dentin sample blocks from each group underwent ultrasonic cleaning and AFM scanning (SPM9700; Shimadzu, Kyoto, Japan) with a scan area of 5×5 μm^2^ and a frequency of 1 Hz. The Shimadzu SPM-9700 software (Shimadzu, Kyoto, Japan) was used to analyze the images, and the surface roughness of each sample was calculated. Finally, statistical analyses were conducted.^36^

#### SEM observation

Following AFM analysis, enamel and dentin sample blocks from each group were sectioned and polished into 4×4×2-mm^3^ blocks. Following ultrasonic cleaning, the samples were sequentially dehydrated using ethanol solutions at 25, 50, 75, and 95% concentrations. Subsequently, the samples were placed in a vacuum gold plating machine and coated with gold.^37^ SEM (Apreo 2; Thermo Fisher Scientific, Brno, Czech) was used to observe the microstructure of the enamel and dentin at 5,000- and 40,000-× magnifications, with a working distance of 10±1 mm and an accelerating voltage of 20 kV.

#### EDS examination

After conducting the experimental procedures for SEM, specific areas within the samples were selected for analysis. Line scanning was performed using EDS (X-MaxN; Oxford instrument, Oxford, UK).^36^ The collected data were compared and analyzed after the creation of a smooth line diagram to facilitate the detection of the content and distribution of calcium (Ca), phosphorus (P), and carbon (C) in enamel and dentin.

#### TMR examination

Finally, the samples were cut into 1 mm-thick slices perpendicular to the window opening surfaces using a slow-cutting machine (Minitom; Struers, Copenhagen, Denmark) at a speed of 100 rpm. All thin slices were polished to a 100-120-μm thickness with 1500 grit carbide polishing paper (Yu Ying, Foshan, China).^34^ A vernier caliper (Mitutoyo, Tokyo, Japan) was subsequently employed to verify that the final thickness fell within the specified range of 110-120 μm. Each slice was then affixed to a TMR imaging slide (Konica Minolta, Tokyo, Japan) and microradiographed alongside an aluminum calibration step wedge with 11 steps for 30 minutes, using a monochrome CuK X-ray source (voltage: 20 kV, current: 20 mA, working distance: 40 cm). Photographic plates were developed and fixed according to standard procedures. Images were captured using a transmitted light microscope with a 20 × objective (Zeiss, Oberkochen, Germany), which was equipped with a CCD camera (Canon, Tokyo, Japan) and connected to a computer (TOSHIBA, Tokyo, Japan). In total, three non-overlapping areas were selected for each sample, and mineral loss and lesion depth were analyzed using TMR Software 2006 (Inspektor Research Systems, Amsterdam, Netherlands).^38^

#### Statistical analysis

Data were analyzed using SPSS 26.0 (IBM; Armonk, New York, USA). The Shapiro-Wilk and Levene tests were employed to assess the normality of the data distribution and the homogeneity of variance, respectively. Roughness data showed a normal distribution and satisfied the homogeneity of variance assumption. Thus, ANOVA was used to analyze the results, with LSD analysis conducted for pairwise comparisons. Conversely, the data on mineral loss and lesion depth failed to meet the criteria for normal distribution and homogeneity of variance, prompting the Kruskal-Wallis H test for statistical analysis. Finally, the results are shown as median ± interquartile range, with a significance level at α=0.05.

## Results

### Surface roughness

**Figure 2:**
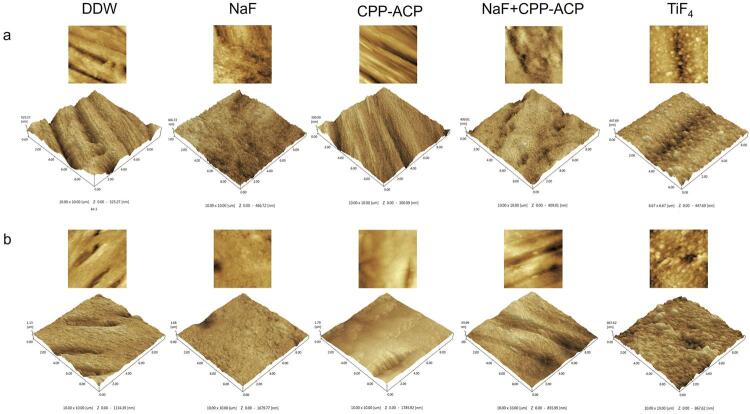
(a) Representative AFM images of enamel and (b) dentin surfaces in each group.

AFM assessed the surface roughness of the samples ( Figure 2a , b). The enamel surfaces in the DDW and CPP-ACP groups showed more significant signs of acid erosion and lesser smoothness. In contrast, the NaF + CPP-ACP and NaF groups showed more uniform and densely clustered deposits on the enamel and dentin surfaces. However, the TiF_4_ group showed globular deposits with a larger diameter.

Table 2 shows the average surface roughness for each group. The enamel samples in the NaF + CPP-ACP group showed the lowest surface roughness at 47.22±15.10 nm, which was significantly lower than that of the other groups (p<0.05). However, no significant differences occurred between the remaining groups (p>0.05). In the dentin samples, the TiF_4_ group showed the lowest surface roughness at 91.57±14.44 nm, followed by the NaF + CPP-ACP group at 110.13±15.46 nm. The difference in surface roughness between the TiF_4_ and NaF + CPP-ACP groups was statistically insignificant (p>0.05), whereas the differences between the TiF_4_ group and the other three groups were statistically significant (p<0.05).

**Table 2:** Quantitative analysis of surface roughness of enamel and dentin in each group.

	Enamel	Dentin
	**Roughness (Ra/nm)**	**Roughness (Ra/nm)**
DDW	62.43±20.13^a^	117.57±27.26^a^
NaF	64.22±12.36^a^	111.47±19.85^a^
CPP-ACP	72.24±28.04^a^	119.39±28.59^a^
NaF + CPP-ACP	47.22±15.10^b^	111.47±19.85^a^
TiF_4_	65.92±7.66^a^	91.57±14.44^b^
p value	p<0.05	

All values are shown as means ± SD. Different letters indicate statistically significant differences.

### Micromorphology

**Figure 3:**
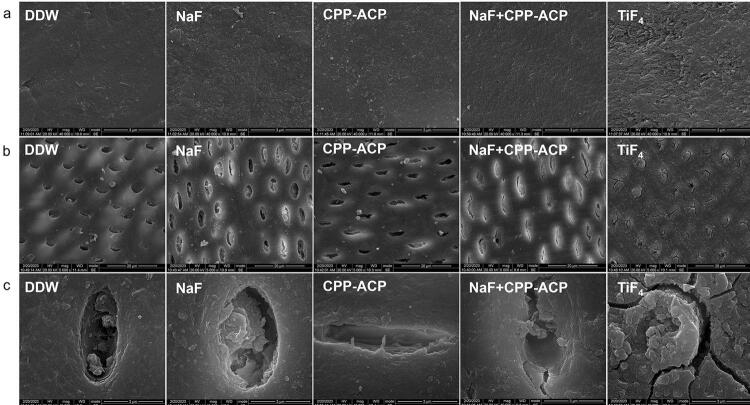
(a) Representative SEM image of enamel (5000 ×), (b) dentin (5000 ×), and (c) dentin (40000 ×) in each group.

The SEM analysis examined the surface structure characteristics of enamel samples, focusing on smoothness; roughness; and the volume, density, and morphology of deposits. In the enamel samples ( Figure 3a ), the surface morphology of all the material treatment groups changed when compared to the DDW group, and a layer of mineral deposition appeared. In the NaF + CPP-ACP group, the sample surface had a more regular, smoother, and more homogeneous appearance than the others. Notably, the sediment particles in the TiF_4_ group showed larger diameters and a densely packed structure, resulting in a less smooth surface.

For dentin samples, evaluation focused on the integrity and deposits of dentin tubules and the arrangement and continuity of dentin collagen fibers. In dentin groups ( Figure 3b , c), demineralization in the DDW group partially removed the deposit in the occluded dentin tubules (partially opening them). A similar situation occurred in the CPP-ACP group. However, the dentin tubules in the NaF + CPP-ACP and NaF groups had a limited degree of openness, with many deposits still present in most dentin tubules, and the dentin in TiF_4_ group formed a rough and uneven layer on the surface. All dentin tubules showed completely blockage.

### Elements distribution

**Figure 4:**
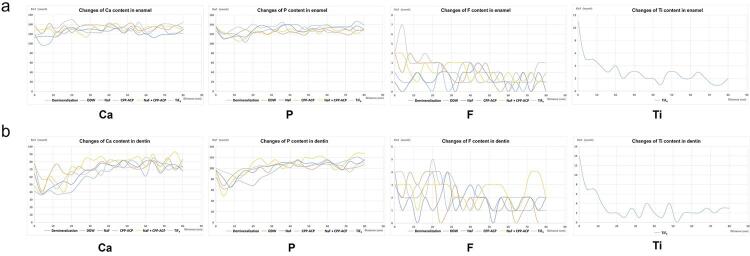
(a) The changes of Ca, P, F, and Ti content in irradiated enamel and (b) dentin at varying depths.

The EDS experiment collected elemental counts at various depths from the sample surface. The EDS results ( Figure 4a , b) showed that the Ca and P content in the enamel and dentin in all treatment groups tended to decrease and then increase with depth, whereas the DDW group showed a gradual increase with depth. The Ca and P of the TiF_4_ group reverted to levels resembling those in the deeper layers at a 10-μm depth, at 20-μm one for the NaF and NaF + CPP-ACP group, a 35-μm one for the CPP-ACP group, and at 40-μm one for the DDW group. As for the variation of F content, the enamel samples showed the deepest depth of entry of F in the NaF + CPP-ACP group; about 20 μm, compared to about 12 μm in the TiF_4_ group and only 5 μm in the NaF group. No significant increase of F occurred in the dentin sample surface. Analyzing the content of Ti on the surface of the TiF_4_ group, the enamel and dentin surfaces were rich in Ti at 20 μm and 15 μm, respectively.

Overall, the EDS results showed that TiF_4_ significantly enriches Ti elements on the surfaces of enamel and dentin samples, thereby substantially reducing the depth of mineral loss. In contrast, NaF + CPP-ACP primarily prevented mineral loss by the action of F elements.

### Mineral loss and lesion depth

**Figure 5:**
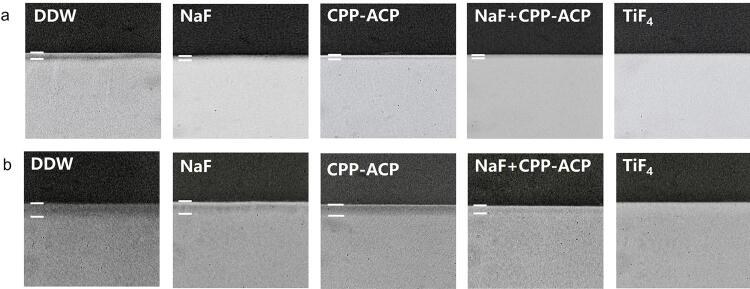
(a) TMR images of demineralized enamel and (b) dentin.

TMR results ( Figure 5a , b) showed that significant demineralized layers could occur on the enamel and dentin surfaces of all groups except the TiF_4_ one, all treatment groups showed a lower degree of demineralization than the DDW group. Table 3 quantitatively analyzed mineral loss and lesion depth for each group: in the enamel samples, the mineral loss in the DDW group totaled 660 ± 600Vol% × µm, and all treatment groups except the CPP-ACP group significantly differed from the DDW group, with the TiF_4_ group having the least mineral loss (230±107.5Vol% × µm), followed by NaF + CPP-ACP (345 ± 80Vol% × µm) and NaF (455±175Vol% × µm). TiF_4_ showed the lowest lesion depth at 6.55±7.0µm, and all treatment groups significantly differed from the DDW group (p<0.001). Among the dentin samples, TiF_4_ had the lowest mineral loss (205±135 Vol% × µm), followed by NaF + CPP-ACP, and all treatment groups significantly differed from the DDW group, except for the CPP-ACP group. The lesion depth in the TiF_4_ group (9.75±3.6 µm) was much lower than that of other groups but significantly indifferent from that of the NaF + CPP-ACP group (21.05±28.8µm).

**Table 3:** Quantitative analysis of mineral loss and lesion depth of demineralized enamel and dentin.

	Enamel		Dentin	
	**Mineral loss**	**Lesion depth**	**Mineral loss**	**Lesion depth**
	**(Vol% × μm)**	**(μm)**	**(Vol% × μm)**	**(μm)**
DDW	660±600^a^	44.80±19.1^a^	1175±340^a^	62.30±17.4^a^
NaF	455±175^cd^	12.85±2.5^bc^	380±730^cd^	23.60±43.3^cd^
CPP-ACP	755±430^ad^	17.45±15.5^c^	830±195^ad^	50.45±6.9^ad^
NaF + CPP-ACP	345±80^bc^	11.00±2.5^bc^	290±280^bc^	21.05±28.8^bc^
TiF_4_	230±107.5^b^	6.55±7.0^b^	205±135^b^	9.75±3.6^b^
p value	p<0.001			

All values are presented as means ± SD. The different letters indicate statistically significant differences.

## Discussion

According to previous research,^11^ radiation exposure at a dose of 50 Gy altered the crystal structure of enamel and degraded the microstructure of dentin. Latest research also indicated no difference in the effects of single total-dose radiation or fractionated radiation on the enamel structure.^39^ Therefore, this study selected enamel and dentin samples that received a single 50 Gy radiation dose and divided them into five groups: the DDW, the NaF, the CPP-ACP, the NaF + CPP-ACP, and the TiF_4_ groups. To simulate clinical practice, this study applied all its materials to the surface of the samples with micro brushes, enabling contact for 30 minutes.^40^The experimental results indicated that the TiF_4_ and NaF + CPP-ACP treatments could prevent the demineralization of irradiated dental hard tissues, corroborating its hypothesis. Notably, TiF_4_ better prevented demineralization.

Demineralization and remineralization of dental hard tissues in the oral cavity occur in alternating cycles, but under pathological conditions, demineralization tends to outpace remineralization.^41^ Dental hard tissue demineralization is due to the metabolic acid production by bacteria adhering to the tooth surface, which decreases the local pH and dissolves calcium and phosphate ions, ultimately destroying the hydroxyapatite structure and forming caries.^42^ Therefore, controlling tooth demineralization after radiotherapy is a priority in preventing RRC. Currently, the application of fluoride during HNC radiotherapy is the main recommended treatment for RRC.^43 , 44^ The anti-caries mechanism of NaF includes generating calcium fluoride (CaF_2_) with calcium ions on the surface of the tooth, which can neutralize the acid reaching the tooth surface; providing a buffer against acid erosion and acting as a reservoir for calcium and fluoride ions; and combining fluoride ions with apatite on the surface of the tooth to form fluorapatite, which better resists acid erosion.^45 , 46^ In this in vitro study, the NaF group formed uniform deposits on enamel and dentin surfaces, showing a certain ability to prevent demineralization. CPP-ACP can release phosphate and carboxylate ions, which combine with calcium and phosphorus ions to form stable amorphous calcium phosphate clusters and gradually increase the concentration of calcium and phosphate ions in saliva, preventing demineralization and enhancing remineralization.^47 , 48^ The CPP-ACP group showed no better resistance to demineralization than other treatment groups in this experiment. This might be attributed to the larger particle sizes in the amorphous structure deposits formed in this group and the damage and disintegration of dental tissue due to radiation^49^ as the deposits could only loosely adhere to the tooth surface and were thus prone to dissolution in an acidic environment.

However, CPP-ACP can interact with fluoride ions to form an amorphous calcium-fluoride-phosphorus complex, slowly releasing calcium, phosphate, and fluoride ions under low pH conditions and improving the ability of the teeth to resist demineralization.^25^ This explains the stronger association of NaF and CPP-ACP than the two alone in this experiment. These results resemble those in some previous studies. Sim, et al.^50^ (2018) noted that in patients undergoing radiotherapy for HNC, combining CPP-ACP provided better caries prevention than fluoride alone. Another study confirmed that remineralization toothpaste with CPP-ACP increased the bioavailability of calcium and phosphorus ions, balanced the demineralization/remineralization process, and positively prevented the formation of cervical caries.^51^

Although NaF in combination with CPP-ACP better prevented demineralization, the collapse of dentinal tubules after radiotherapy prevented the penetration of large-sized deposits into the tooth and hindered the depth of CaF_2_ deposition by NaF. Therefore, NaF in combination with CPP-ACP may fail to optimally prevent RRC. TiF₄, as other fluorides, promotes remineralization and prevents demineralization by facilitating the action of fluoride ions. Its low pH value induces an acidic environment that partially dissolves hydroxyapatite on the tooth surface, facilitating the diffusion of fluoride ions into deeper layers.^52^Moreover, it can form an acid-resistant layer rich in titanium oxide and hydrated titanium phosphate on the surface of dental hard tissues.^53 , 54^As the concentration of TiF4 increases, the resulting titanium-rich layer becomes thicker and denser, serving as an acid-resistant barrier against enamel erosion.^21 , 55^This enables dentin that is unable to form CaF2 deposits due to radiation damage to resist acid erosion by an alternative method. The AFM and SEM results in this study showed that even after demineralization, the enamel and dentin surfaces in the TiF_4_ group still had more obvious deposits that differed from those of the NaF group, and the EDS results showed Ti elemental aggregated in dentin surfaces of the TiF_4_ group, forming a titanium-rich layer. According to Tveit, et al.^56^ (2009), the titanium-rich layer could still remain on the dentin surface after three weeks of using TiF_4_ preparations in the mouth, indicating its long-lasting and effective anti-acid effect. The results of TMR and EDS in this study support the superiority of TiF_4_ over NaF in preventing demineralization. This agrees with Comar, et al.^21^(2018), who indicated that TiF_4_ varnish more effectively prevented enamel demineralization than a NaF varnish.

The experimental design falls subject to certain limitations. Although this study chose crack- and spot-free bovine incisors of similar size and ensured a sufficient sample size and a strict randomization to minimize bias, this study fail to record the baseline microhardness and surface roughness of its sample due to equipment failure. This methodological flaw may have caused differences in the initial mineral content of the samples. Future studies should improve their design to ensure data integrity. While the *in vitro* model isolated variables and provided controlled conditions, it lacked the complexity of the *in vivo* environment. For instance, this study ignored the absence of saliva and its dynamic interactions with dental tissues and materials. Future investigations could incorporate a more physiologically relevant model that better mimics the oral cavity. Additionally, the 30-minute application in this study may be suboptimal as prolonged or more frequent treatments might yield different outcomes. Furthermore, despite its excellent remineralization-promoting^11^ and demineralization-preventing effects, TiF_4_ may cause mucosal irritation and discomfort due to its low pH. This requires strict isolation of teeth from adjacent tissues during treatment, increasing the complexity of clinical procedures. Moreover, according to de Souza,^57^ TiF_4_ has no antibacterial ability, so further clinical trials must assess the actual prevention of RRC.

## Conclusions

TiF_4_ significantly affected the prevention of *in vitro* demineralization of irradiated enamel and dentin. NaF, in combination with CPP-ACP, more effectively prevented demineralization than the two by themselves. This experiment provides new ideas to prevent and treat RRC.
